# A prospective clinical study to evaluate the performance of zirconium dioxide dental implants in single-tooth edentulous area: 3-year follow-up

**DOI:** 10.1186/s12903-018-0636-x

**Published:** 2018-11-01

**Authors:** Kai-Hendrik Bormann, Nils-Claudius Gellrich, Heinz Kniha, Sabine Schild, Dieter Weingart, Michael Gahlert

**Affiliations:** 1 Hannover Medical School, Hannover, Germany; 2 Dental Clinic, Bormann Oralchirurgie am Hafen, Hamburg, Germany; 3 Dental Clinic Kniha Gahlert, Munich, Germany; 4 High Tech Research Center, University Hospital Basel, Basel, Switzerland; 5 Klinikum Stuttgart, Katharinenhospital, Stuttgart, Germany

**Keywords:** Dental implant, Zirconium dioxide, Ceramic implant, Zirconia, ZrO2, ZLA

## Abstract

**Background:**

Traditionally, dental implants have been made from titanium or titanium alloys. Alternatively, zirconia-based ceramic implants have been developed with similar characteristics of functional strength and osseointegration. Ceramic implants offer advantages in certain settings, e.g. in patients who object to metal dental implants. The aim of this study was to investigate the mid-term (36 months) clinical performance of a ceramic monotype implant in single-tooth edentulous area.

**Methods:**

This was a prospective, open-label, single-arm study in patients requiring implant rehabilitation in single-tooth edentulous area. Ceramic implants (PURE Ceramic Implant, Institut Straumann AG, Basel, Switzerland) with a diameter of 4.1 mm were placed following standard procedure and loaded with provisional and final prostheses after 3 and 6 months, respectively. Implant survival rate and implant success rate were evaluated and crestal bone levels were measured by analysing standardized radiographs during implant surgery and at 6, 12, 24 and 36 months.

**Results:**

Forty-four patients received a study implant, of whom one patient withdrew consent after 3 months. With one implant lost during the first 6 months after surgery, the implant survival rate was 97.7% at 6 months. No further implants were lost over the following 30 months, and 3 patients were lost to follow-up during this time frame. This led to a survival rate of 97.5% at 36 months.

Six months after implant surgery 93.0% of the implants were considered “successful”, increasing to 97.6% at 12 months and remaining at this level at 24 months (95.1%) and 36 months (97.5%).

Bone loss was most pronounced in the first half-year after implant surgery (0.88 ± 0.86 mm). By contrast, between 12 and 36 months the mean bone level remained stable (minimal gain of 0.06 [± 0.60] mm). Hence, the overall bone loss from implant surgery to 36 months was 0.97 (± 0.88) mm.

**Conclusions:**

In the follow-up period ceramic implants can achieve favourable clinical outcomes on a par with titanium implants. For instance, these implants can be recommended for patients who object to metal dental implants. However, longer term studies with different edentulous morphology need to confirm the present data.

**Trial registration:**

Registered on www.clinicaltrials.gov: NCT02163395.

## Background

For over 40 years, dental implants have been used for the replacement of compromised, lost or missing teeth, following the principles of osseointegration as reported by Brånemark and Adell [1, 2]. While clinical studies have proved the longevity and success of titanium dental implants [3–8], the aesthetics of titanium implants can be challenging, especially in the maxillary incisor area when the dark grey colour of the metal becomes visible. Furthermore, there is a risk of hypersensitivity reaction in patients who are allergic to metals or patients might demand metal-free restorations [9].

Ceramic (zirconia [ZrO_2_]) implants were developed to solve the limitations associated with titanium-based alloys. For instance, the ceramic implant is a good option when it comes to aesthetics, as the shade of zirconia is similar to the colour of a tooth. Like titanium, zirconia also has excellent mechanical, biocompatible and osseointegration properties [10–17] with the potential to serve as a successful implant material with high survival and success rates [18, 19] and only small bone losses [20–22]. However, it is difficult to make a consensus regarding the efficacy of ceramic implants due to the variations in ceramic implants and study designs [23].

It has already been shown that certain ceramic implants can achieve clinical outcomes like those of titanium implants after 12 months of follow-up [24]. The present study aims to investigate the safety and clinical performance of this type of ceramic implant concerning survival and success rates, as well as hard and soft tissue parameters after 36 months in the same cohort.

## Methods

The study was a prospective 36-month follow-up of an open-label, single-arm study. In the meantime, the study has been extended to 10 years, with planned patient visits at 5 and 10 years after implant surgery. The materials and methods of the study were published previously [24] and are briefly set out below. The study is registered under www.clinicaltrials.gov (study no. NCT02163395).

### Patients

Patients were selected according to predefined eligibility criteria. The main inclusion criterion was a single-tooth edentulous area in the mandible (Fédération Dentaire Internationale [FDI] tooth positions 36 to 46) or maxilla (FDI tooth positions 16 to 26), and a natural tooth had to be distally and mesially adjacent to the implant site. The implant positions had to have mainly healed (at least 8 weeks after extraction) and any bone augmentation with autogenous bone had to have been performed at least 3 months prior to implant surgery. Exclusion criteria included different systemic diseases (e.g., uncontrolled diabetes, mucosal disease, untreated periodontitis, gingivitis, or endodontic lesions), smoking more than 10 cigarettes a day, probing pocket depth of ≥4 mm on a tooth immediately adjacent to the implant site, severe bruxing or clenching habits, inadequate oral hygiene and history of local irradiation therapy. Additional secondary exclusion criteria were applied at or after surgery: lack of implant primary stability, inappropriate implant position for prosthetic requirements, major simultaneous augmentation procedures at surgery, and X-ray not showing the implant from first bone contact to apical tip. The complete list of the eligibility criteria can be found in the first publication of the study [24].

### Study device

Ceramic implants (PURE Ceramic Implant, Institut Straumann AG, Basel, Switzerland) with a regular diameter (4.1 mm) were placed in this study. These ceramic implants represent monotype one-piece zirconia implants with already integrated abutments into the implant design, avoiding a microgap between implant and abutment. They are made of yttria-stabilized zirconia (Y-TZP) and are available in 4 different lengths and 2 different abutment heights. The implant is based on the features of the Straumann® Tissue Level Standard Plus and Straumann® Bone Level Implants (Institut Straumann AG, Basel, Switzerland) and has a sandblasted, large-grit, acid-etched surface like the SLA surface present in titanium implants. Further details are provided in the first publication of the study [24].

### Surgical and restoration procedure

The implant surgeries were performed by KHB, HK, SS and MG at baseline following standard procedures. Sutures were removed 7 to 14 days after implant surgery, and provisional and final prostheses were placed 11 to 13 weeks and 24 to 28 weeks, respectively, after the implants had been placed. No particular prosthetic procedure was defined in the study protocol. Due to the monotype design of the ceramic implant, lithium disilicate glass–ceramic or zirconium dioxide ceramic crowns were inserted in most cases. Permanent glass ionomer luting cement was used to cement the crowns directly onto the implant abutment. Dental floss was used to remove eventual cement remnants.

The patients attended follow-up visits 12, 24 and 36 months after surgery. Additional follow-up visits are planned 5 and 10 years after implant surgeries. Further details are provided in the first publication of the study [24].

### Efficacy evaluations

This is a report of secondary outcomes at 24 and 36-months after implant surgery of:

- Implant survival
- Implant success
- Mean (distal and mesial) bone levels

#### Implant survival

Implants that were still in place 6, 12, 24 and 36 months after implant surgery were considered as surviving implants.

#### Implant success

Implant success was defined according to these previously described criteria [25]:

- Absence of pain, foreign body discomfort or dysaesthesia
- Absence of recurrent peri-implant infection with suppuration
- Absence of implant mobility
- Absence of continuous peri-implant radiolucency.

Failed implants were considered as not successful.

#### Bone levels

**Fig. 1:**
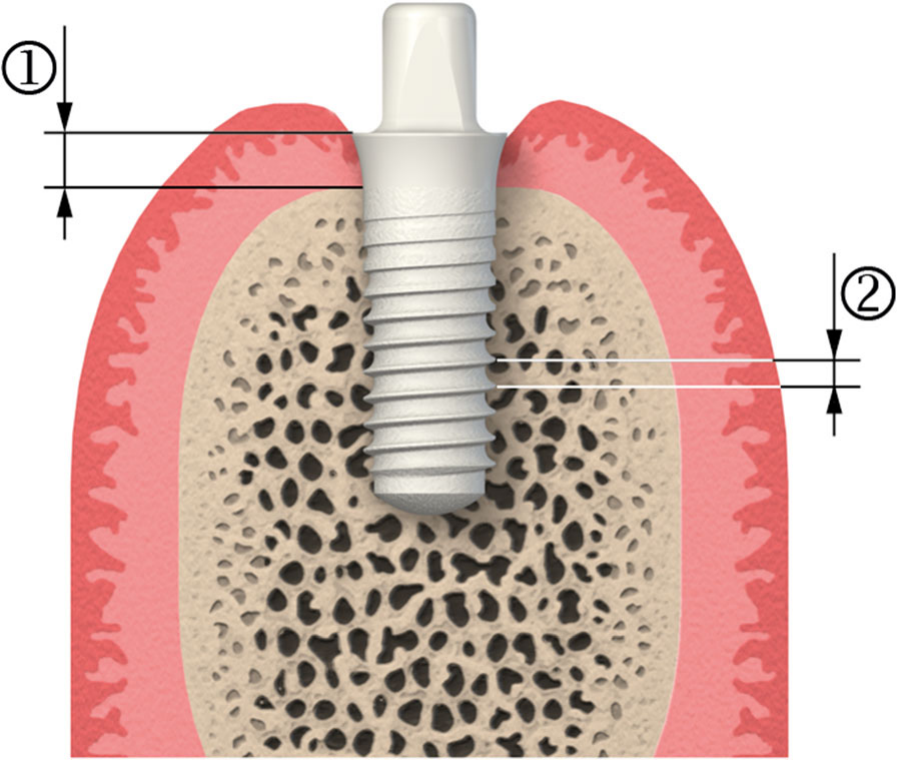
Illustration of the bone level measurements. (1) Implant shoulder to first implant-to-bone contact. (2) Distance between the threads of the implant

Standardized radiographs were taken at baseline (day of implant surgery) as well as 6, 12, 24 and 36 months after implant surgery. Vertical bone level assessment was performed by measuring from the implant shoulder to the first visible bone contact on the implant. Measurements were made at the distal and mesial aspects of each implant, and an average was calculated (Fig. 1). Distortions (changes on the radiographs from the true implant dimensions) were accounted for by normalizing the measurements to the known thread pitch of the implants. All measurements were analysed by an independent specialist.

#### Plaque index, sulcus bleeding index and Oral hygiene instructions

Plaque and sulcus bleeding indices were assessed at the implant site and in the adjacent teeth investigating if there was plaque and bleeding, respectively (yes/no).

All patients were given oral hygiene instructions during each study visit. Further details about efficacy evaluations are provided in the first publication of the study [24].

### Complications

Adverse events (AEs) and device deficiencies (DDs) monitoring was performed throughout the study. The patients were asked at every visit if they experienced any complication (like pain, bleeding, etc) since the last visit.

### Statistical analysis

Data management was performed using DMSys data management software (Version 5.3), Sigmasoft International, and statistical analysis using IBM SPSS statistical software (Version 21, IBM).

In short, descriptive summary statistics were calculated for all documented parameters, including mean values and standard deviations.

For survival and success rates, patients who were lost to follow-up during the 36-month follow-up period were excluded. At each follow-up visit survival and success rates were calculated by dividing implants still in place and patients meeting all the success criteria, respectively, by the patient population at this follow-up visit. 95% confidence intervals (CIs) were calculated using the modified Wald method.

Mean bone level changes were calculated by subtracting the average bone levels at 6, 12, 24 and 36 months from the average bone level at baseline (day of implant surgery). In other words, positive changes represent bone gain between baseline and 6, 12, 24 and 36 months, respectively, whereas negative changes represent bone loss.

The intention to treat (ITT) population includes all patients who received implant treatment and underwent at least one measurement after surgery, regardless of any major protocol deviations or premature termination. The per protocol (PP) population includes all patients who received an implant and completed the 36-month follow-up period without major protocol deviations. The safety analysis set (SAS) includes all patients who received implant treatment.

Further statistical analysis details are provided in the first publication of the study [24].

## Results

### Patients

In October 2011, the first patient was enrolled. Four years later (in October 2015), the last patient completed the 36-month study period.

**Fig. 2:**
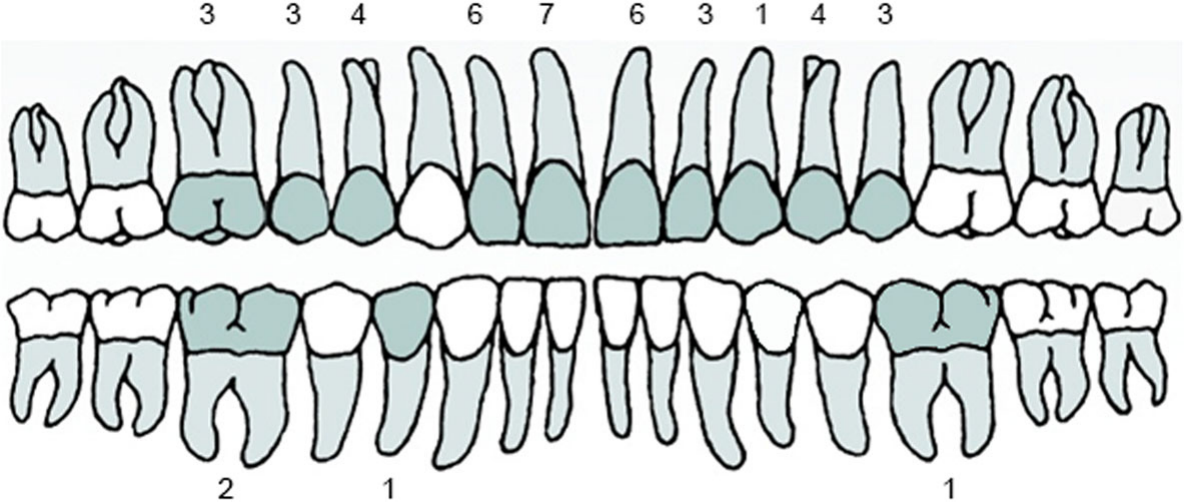
Distribution of implants placed in the study. Number of implants placed at each tooth position. *N* = 44. N: Total number of implants placed

Forty-six patients were screened, of whom 44 (17 males and 27 females) subsequently received the study implant and were analysed according to the ITT data set. Patients were on average 48 ± 14 years old (median: 49 years, range: 18 to 78 years). Most of the implants were placed in the maxilla (40 implants; 90.9%), whereas only 4 implants (9.1%) were placed in the mandible (Fig. 2). Bone augmentation was necessary in 31.8% of the cases (14 out of 44 sites); all of them in the aesthetic zone and at the time of surgery. All were considered as non-major augmentations.

Of these 44 patients, 5 terminated the study early and were therefore excluded from the PP analysis. They were either lost to follow-up (3 patients; after 6, 12 and 24 months, respectively), withdrew consent (1 patient; after 3 months) or experienced an AE leading to the implant loss (1 patient; after 6 months). A further 8 patients were excluded from the PP analysis due to major protocol deviations. One patient, who terminated the study early, experienced a major protocol deviation, as a provisional crown had not been placed.

In addition, time window deviations were observed for many patients. These were considered “minor protocol deviations”, with no consequences for the data set allocation.

Hence, in total, 13 patients in the ITT population had to be excluded from the PP population, and efficacy analysis according to PP analysis was possible in 31 patients.

### Clinical performance evaluations

#### Implant survival and success

**Fig. 3:**
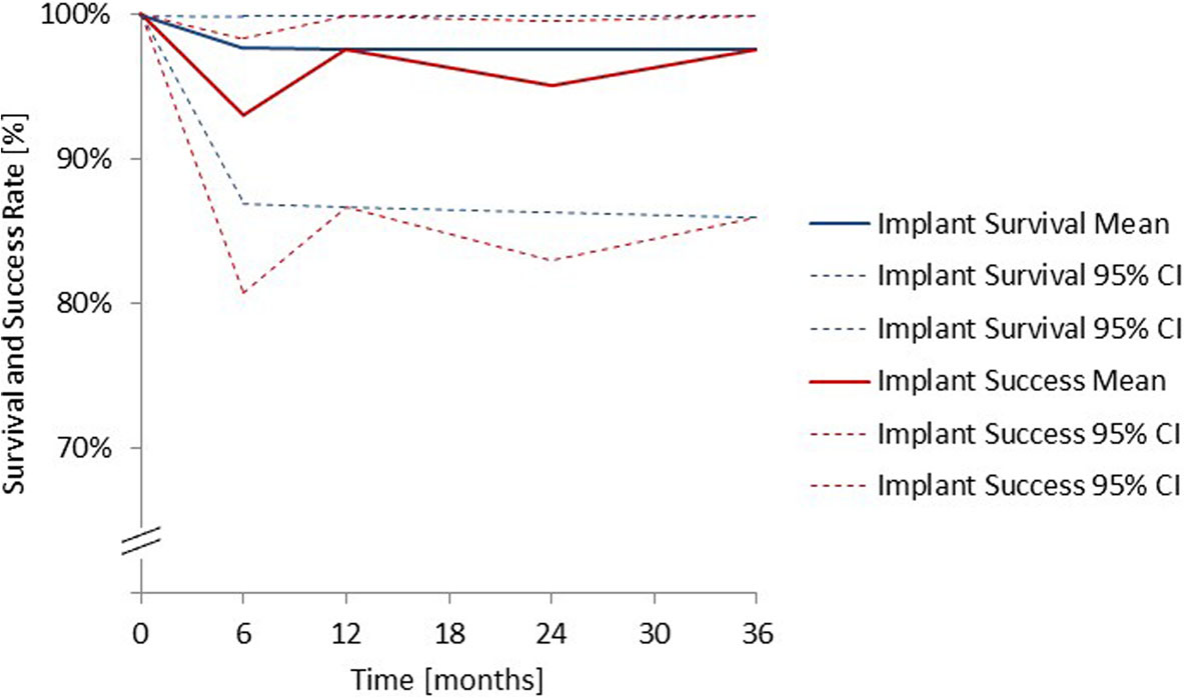
Survival and success rate analysis. Scaling of the Y-axis 70% to 100%. ITT population (*n* = 44). n (0 m) = 44; n (6 m) = 43; n (12 m) = 42; n (24 m) = 41; n (36 m) = 40. Patients lost to follow-up during the 36-month follow-up period were excluded. CI: Confidence interval; ITT: Intention to treat; m: Months; n: Number

The implant survival and success rates were derived from the ITT data set, with patients who were lost to follow-up being excluded from the analysis. The only implant loss during the 36-month follow-up period occurred before final implant loading. Three patients were lost between 6 and 36 months. These led to a survival rate of 97.7%, with 95% CIs of 86.9% to > 99.9% within the first 6 months after implant surgery and stayed at this level until 36 months (Fig. 3).

During the 36-month follow-up period, a total of 4 implants were considered as “not successful”. One patient lost the implant before month 6. At 6 months, 2 patients experienced a mucositis, one of whom also showed continuous radiolucency around the implants. At 24 months, one patient was considered as “not successful” as he/she experienced a mucositis and also pain, foreign body discomfort or dysesthesia. At 12 and 36 months, the only implant considered as “not successful” was the lost implant. Hence, the success rate is equal to the survival rate at these two visits. Overall, the implant success rate was greater than 90% throughout the 36-month follow-up period and, at 12, 24 and 36 months, greater than 95% (Fig. 3).

#### Bone levels

**Fig. 4:**
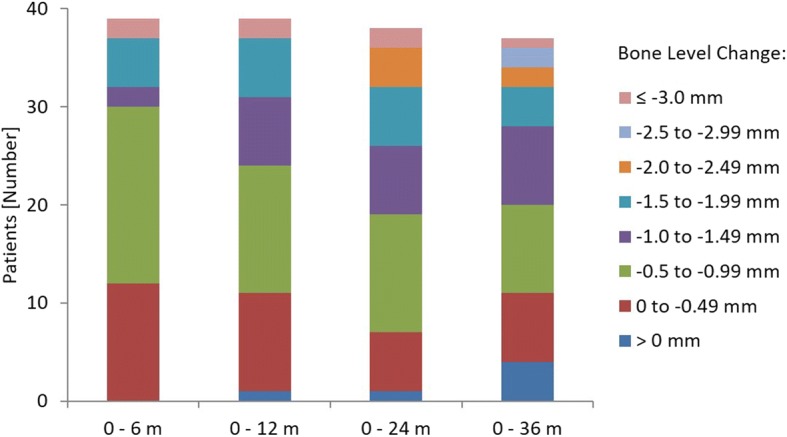
Categorized bone level changes. ITT population (*n* = 44). n (0–6 m) = 39; n (0–12 m) = 39; n (0–24 m) = 38; n (0–36 m) = 37. Missing values were excluded from the analysis. ITT: Intention to treat; m: Months; n: Number

**Fig. 5:**
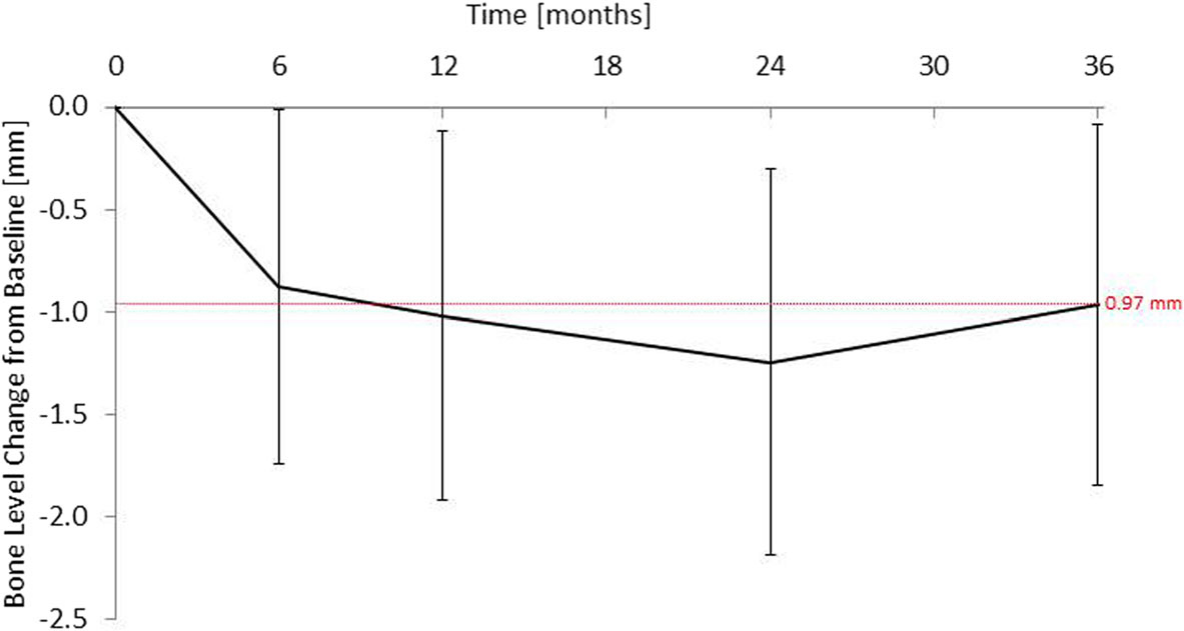
Bone levels (mean ± SD) from implant surgery to 36-month follow up visit. ITT population (*n* = 44). n (0–6 m) = 39; n (0–12 m) = 39; n (0–24 m) = 38; n (0–36 m) = 37. Missing values were excluded from the analysis. ITT: Intention to treat; m: Months; n: Number; SD: Standard deviation

Bones levels were derived from the ITT data set. Missing data were not calculated. Over the 36 months 54.1% of implant sites (*n* = 20) showed bone loss of less than 1.0 mm or bone gain (Fig. 4). Bone loss in general was most pronounced in the first half-year after implant surgery (0.88 ± 0.86 mm). By contrast, between 12 and 36 months the mean bone level remained stable (minimal gain of 0.06 [± 0.60] mm). Hence, the overall bone loss between implant surgery and 36 months was 0.97 (± 0.88) mm. The bone levels at 6, 12, 24 and 36 months were significantly different from baseline at implant surgery (Fig. 5). The ranges of bone losses were 0.1 to 4.4 mm, − 0.2 to 4.6 mm, − 0.2 to 4.7 mm and − 0.8 to 3.0 mm 6, 12, 24 and 36 after the implant surgery, respectively. Note, negative bone losses representing bone gains.

#### Plaque index

**Fig. 6:**
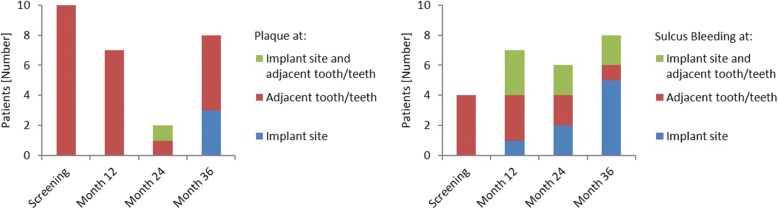
Number of patients with plaque and sulcus bleeding. ITT population (n = 44). ITT: Intention to treat; n: Number

Plaque was observed in 17 (38.6%) patients during the study, mostly at screening (10 occurrences [58.8%]), at month 36 (8 occurrences [47.1%]) and at month 12 (7 occurrences [41.2%]). Until month 24 plaque occurrence was only recorded for the adjacent tooth or teeth, whereas at month 36, 3 of the 8 occurrences were reported for the implant site (Fig. 6).

#### Sulcus bleeding index

Bleeding was detected in 15 (34.1%) patients during the study, mostly at month 36 (8 occurrences [53.3%]), at month 12 (7 occurrences [46.7%]) and at month 24 (6 occurrences [40.0%]). Bleeding occurred either at the implant site, the adjacent teeth or at both locations (Fig. 6).

### Complications

In total, the following 27 (39.7%) AEs out of the 68 AEs that occurred over the 36-month follow-up period were deemed to be related to the study device or study treatment: pain (*n* = 18), bleeding, swelling, inflammation/infection (*n* = 6) and unfulfilled implant success criteria (*n* = 3). None (0%) of the 7 SAEs were deemed to be related to the study device or study treatment.

## Discussion

The prospective, open-label, single-arm study investigated ceramic implants for replacing a single missing tooth. The implant survival and success rates 36 months after implant surgery were both 97.5%. The mean bone loss was most pronounced in the first half-year after implant surgery (0.88 mm) and remained relatively stable between 12 and 36 months, which resulted in an overall bone loss of 0.97 mm at 36 months.

The survival and success rates of 97.5% 36 months after implant surgery described here are comparable with the published literature on ceramic and titanium implants [26, 27], confirming the good clinical performance of ceramic implant. This within the limits of this study, as a single-arm study design was selected for this study.

In a recent systematic review that included 14 studies using zirconia implants, an overall survival rate of 92% (95% CIs: 87% to 95%) after one year of functional loading was calculated [26]. Only 4 of these 14 studies examined a monotype ceramic implant system separately, and these had an average observational period of at least 2 years. The survival rate in these 4 studies after an average of 2 to 5.9 years was between 77.3 and 100% [22, 28, 29]. The authors state that the heterogeneity of the selected studies may be behind the differences in survival rates. On the one hand the examined zirconia implants varied considerably in implant design and surface characteristics while, on the other, the studies differed in their surgical protocols and prosthetic superstructures [26].

In a systematic review that analysed 46 studies with a mean follow-up of at least 5 years in function after the insertion of titanium implants, the overall survival rate of titanium implants supporting single crowns was 97.2% (95% CIs: 96.3% to 97.9%) after 5 years [27], which is comparable to the survival rate evaluated in the current study. This strengthens the results of several animal studies showing that zirconia implants undergo osseointegration similar to [10, 14, 15, 30–36], or even better than [16], titanium implants, and confirms the biocompatibility of zirconia as a dental implant material [37]. Furthermore, various in vivo and in vitro studies investigating soft tissue response around zirconia discovered similar [38] or even superior healing response, less inflammatory infiltrate and reduced plaque adhesion on zirconium oxide discs compared to pure titanium [39–42]. Excellent gingival conditions around monotype ceramic implants were described by Kniha [43]. In addition, like titanium, zirconia is known to have excellent mechanical properties [11, 13, 17].

In contrast to other ceramic implants, the body of the study device features the ZLA surface, a sandblasted, large-grit, acid-etched surface like the SLA surface of titanium implants [24]. Gahlert compared the removal torque of zirconia implants with either machined or sandblasted surfaces placed in mini-pig jaws [44]. The results revealed that a sandblasted surface can display greater stability in bone than machined surfaces, leading to the conclusion that roughening the zirconia implants improves bone anchoring. Oliva compared zirconia implants with 3 different surfaces in a large study: uncoated, coated and acid-etched. Each group consisted of at least 240 implants [28]. The uncoated implants were roughened by mechanical grinding, while the coated implants were roughened, given a bioactive ceramic coating and subsequently sintered. The survival rate for the acid-etched implants was highest, at 97.6% (n: 333, mean: 3.1 years, range: 1 to 4 years), followed by the coated implants, at 93.6% (n: 249, mean: 3.6 years, range: 2 to 5 years) and the uncoated implants, at 92.8% (n: 249, mean: 3.5 years, range: 2 to 5 years).

In the current study bone loss was 0.97 (± 0.88) mm from implant surgery to month 36, which was similar to [22], or slightly less than [29, 45], results reported in other studies with zirconia implants. The first European Workshop on Periodontology [46] considered a bone loss of less than 1.9 mm after 36 months as “successful.” Hence, the bone loss in the current study may be considered as such, all the more so as most of the implants were placed in the maxilla (90.9%) [24]. Marginal bone loss during the first year of loading has been shown to be more pronounced for implants inserted in the maxilla than for those placed in the mandible [29, 45]. The reported bone loss in two studies on zirconia implants (1.63 mm overall and 1.21 mm for single implants after 48 months, and 1.29 mm after 24 months, respectively) [45] was slightly greater than that observed in the current study. As in the study by Borgonovo [45], in this study also bone loss occurred mainly during the first year and stabilized thereafter. Borgonovo assumed that the relatively large bone loss in the first year could be related to bone maturing after surgery and adapting to withstand functional forces [45]. Furthermore, simultaneous augmentation procedures might result in bone loss during the first year of this study – this might be an explanation for bone losses greater than 3 mm after 6, 12 and 24 months at two implants. In this study, the bone loss of 1.02 (± 0.90) mm during the first 12 months after surgery was below the limit of 1.5 mm and considered to be “successful” [46]. As in the study by Borgonovo [45], even a small bone gain was also observed in this study during the follow-up period. In contrast to the previously reported results, in this study bone gain occurred at a later stage (between month 24 and month 36). Borgonovo assumed that bone gain occurred because of new bone trabeculae forming as a result of bone maturation [45].

Most of the AEs that occurred during the 36 months of this study and that were considered to be “related” to the study treatment were experienced during surgery (visit 2), within one week of surgery, or shortly after/in relation to another study intervention. Hence, these AEs can be considered as expected complications following the insertion of dental implants, confirming that there is no safety issue associated with the placement of the ceramic implants.

## Conclusion

The results demonstrate that ceramic implants achieve favourable clinical outcomes in the follow-up; so are survival and success rates as well as bone losses comparable to titanium and other ceramic implants. These implants offer a reliable and successful treatment alternative, especially useful for patients who object to metal and request metal-free implants. However, longer term studies with different edentulous morphology need to confirm the present data.

## Abbreviations

AE: Adverse event
CI: Confidence interval
DD: Device deficiency
FDI: Fédération Dentaire Internationale
ITT: Intention to treat
m: Months
n: Number
PP: Per protocol
SAS: Safety analysis set
SD: Standard deviation
Y-TZP: Yttria-stabilized zirconia
ZrO_2_: Zirconia
